# Endogenous Fungal Endophthalmitis Following Eyebrow Tattooing: A Case Report

**DOI:** 10.7759/cureus.93246

**Published:** 2025-09-26

**Authors:** Nianjia Wang, Jiayi Wu, Xintong Xiang, Qian Zhao, Liang Yao

**Affiliations:** 1 Ophthalmology, The Second Affiliated Hospital of Xi'an Jiaotong University, Xi'an, CHN

**Keywords:** aspergillus fumigatus, botanical dye tattoo, endogenous fungal endophthalmitis, pars plana vitrectomy (ppv), voriconazole

## Abstract

This case report describes a rare instance of bilateral endogenous fungal endophthalmitis in a 50-year-old healthy female patient, following a facial tattooing procedure. Initially misdiagnosed as iritis in the right eye due to presenting symptoms of blurred vision and ocular pain, the patient's condition worsened following treatment with corticosteroids. Ophthalmic examination revealed severe vitreous opacity in the right eye and a yellowish-white lesion in the inferonasal retina of the left eye. Metagenomic sequencing of the vitreous fluid confirmed infection with Aspergillus fumigatus. The patient underwent pars plana vitrectomy with silicone oil tamponade, retinal laser photocoagulation, and intravitreal voriconazole injection in the right eye. Both eyes received multiple intravitreal voriconazole injections, supplemented with systemic antifungal therapy. Postoperatively, the visual acuity in the right eye improved, and the left eye gradually recovered to 20/35. Serial optical coherence tomography follow-up of the left eye documented the progressive detachment of the fungal embolus from the retinal lesion into the vitreous cavity. This case highlights that traumatic cosmetic procedures, such as eyebrow tattooing, can be a potential risk factor for endogenous fungal infection. In cases of atypical uveitis, early etiological investigation is crucial to avoid misdiagnosis and inappropriate treatment. Dynamic imaging provides valuable evidence for assessing the efficacy of antifungal therapy and determining prognosis.

## Introduction

Infectious endophthalmitis is a serious ophthalmologic emergency that can lead to permanent vision loss [1]. It occurs when pathogens invade intraocular structures, triggering a cascade of inflammatory responses. Common causative agents include bacteria and fungi, though viruses and parasites may also cause uveitis [2]. Routes of infection are categorized as exogenous or endogenous; exogenous infection typically results from direct inoculation via trauma or intraocular surgery, while endogenous infection arises from hematogenous spread from a distant infectious focus. Endogenous endophthalmitis is more frequently observed in individuals with immune dysfunction [3]. This article reports a case of bilateral endogenous fungal endophthalmitis in a healthy woman following eyebrow tattoo. One eye was managed with pars plana vitrectomy (PPV), a surgical procedure to remove the vitreous, combined with intravitreal antifungal agents, while the contralateral eye was effectively treated with multiple intravitreal injections of voriconazole.

## Case presentation

**Figure 1:**
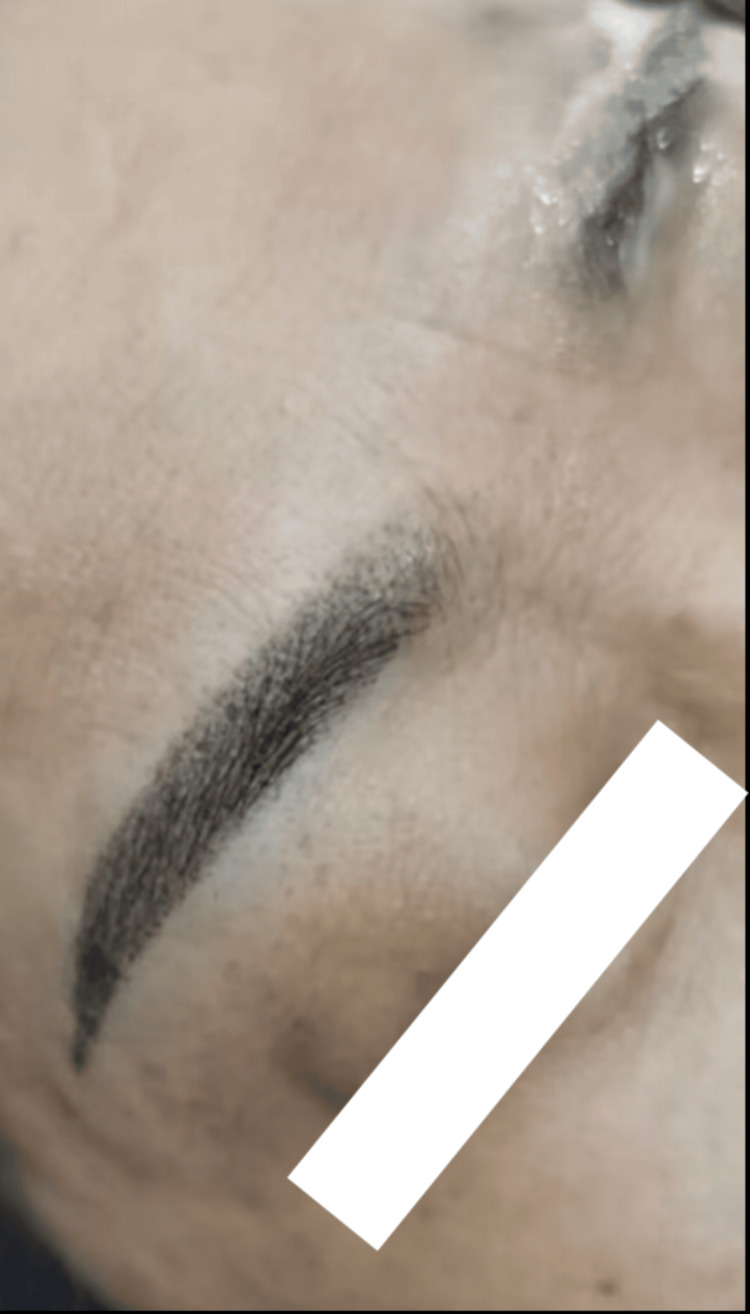
Facial appearance after eyebrow tattooing.

**Figure 2:**
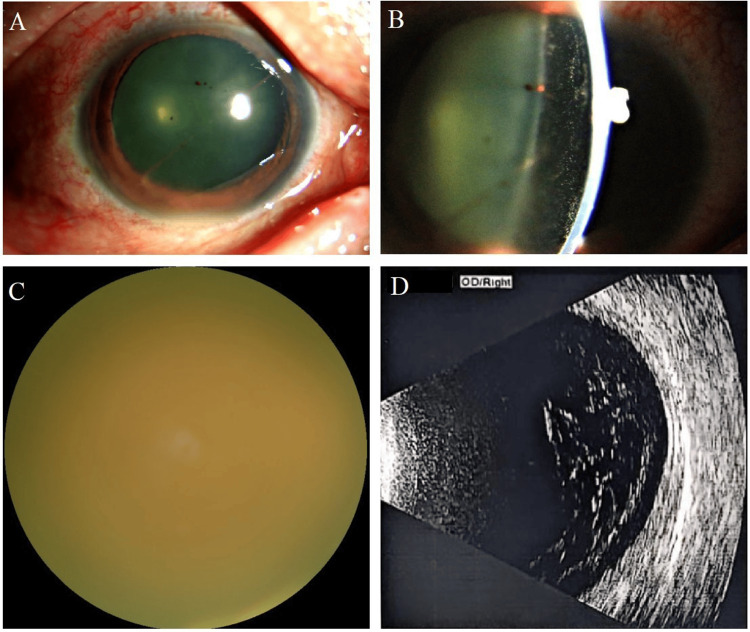
Baseline ophthalmic examination findings of the right eye. (A) Marked ciliary congestion. (B) Slit-lamp examination revealed abundant inflammatory cells and a significant aqueous flare in the anterior chamber. (C) Dense vitreous opacity precluding view of the fundus. (D) Ocular B-scan ultrasound showed extensive vitreous opacities.

**Figure 3:**
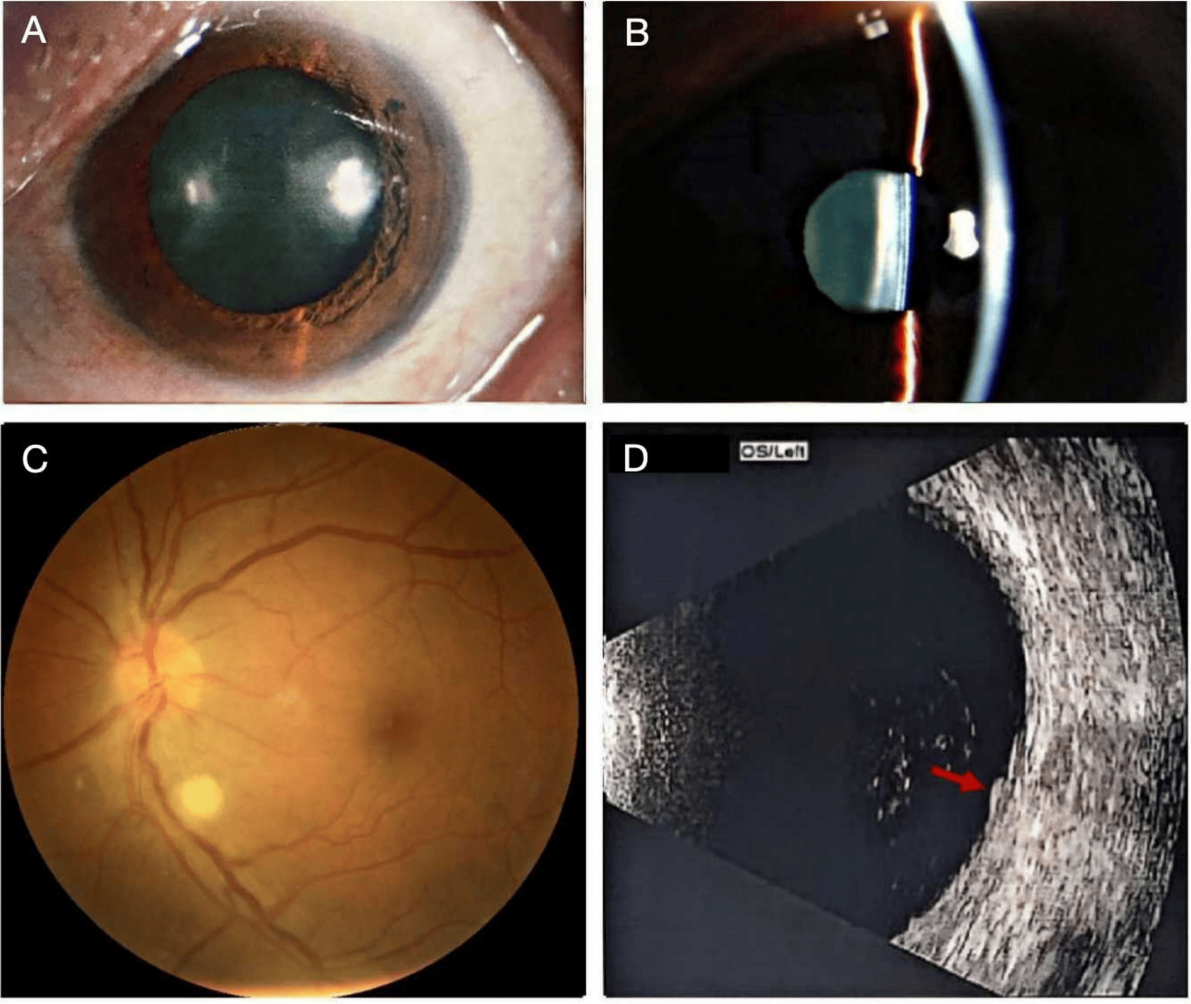
Baseline ophthalmic examination findings of the left eye. (A) No conjunctival or iris hyperemia was observed; the cornea was clear. (B) Slit-lamp examination showed a quiet anterior chamber without cells or flare. (C) A creamy, yellowish-white lesion, approximately 1/3 optic disc diameter in size, was observed adjacent to the inferonasal branch retinal vein. (D) Ocular B-scan ultrasound revealed mild vitreous opacities and a subretinal elevated lesion with high reflectivity inferior to the optic nerve, bulging into the vitreous cavity (red arrow).

A 50-year-old woman with no significant past medical history presented with blurred vision and pain in her right eye for two weeks following eyebrow tattooing (Figure 1). Initially diagnosed with “iridocyclitis” at another institution, she was treated with systemic and topical corticosteroids along with mydriatics for one week, but her symptoms worsened. Upon referral to our hospital, ophthalmic examination revealed the following: in the right eye, visual acuity (VA) was hand motion/10 cm. Marked ciliary congestion was observed, with significant aqueous flare and abundant inflammatory cells in the anterior chamber. Severe vitreous opacity obscured fundus view. Ocular B-scan ultrasound showed extensive vitreous opacities (Figure 2). In the left eye, VA was 20/50. The anterior chamber was quiet, with mild vitreous haze. A creamy yellowish-white lesion, approximately 1/3 optic disc diameter in size, was observed adjacent to the inferonasal branch retinal vein. B-scan ultrasound revealed mild vitreous opacities and an elevated lesion with high reflectivity inferonasal to the optic disc (red arrow). Optical coherence tomography (OCT) showed a poorly demarcated lesion involving the inner retinal layers inferior to the optic disc (Figure 3). Serological testing was negative. A comprehensive workup for other infectious foci (including syphilis, HIV, hepatitis, and tuberculosis) and systemic autoimmune or rheumatologic diseases was unremarkable, with all tested markers within normal limits. Multidisciplinary consultation ruled out infectious foci in the respiratory, gastrointestinal, and oral cavities.

Treatment course

Metagenomic sequencing of vitreous aspirate confirmed infection with Aspergillus fumigatus. Bilateral intravitreal injections of voriconazole (100 μg/0.1 mL) were administered immediately. The right eye subsequently underwent combined phacoemulsification, PPV, silicone oil tamponade, retinal laser photocoagulation, and intravitreal voriconazole injection. Systemic voriconazole was initiated postoperatively with intravenous infusion (200 mg, twice daily), switched to an oral formulation (200 mg) after three days for sequential therapy, gradually tapered, and maintained for six months. During this period, bilateral intravitreal voriconazole injections were repeated every three weeks.

**Figure 4:**
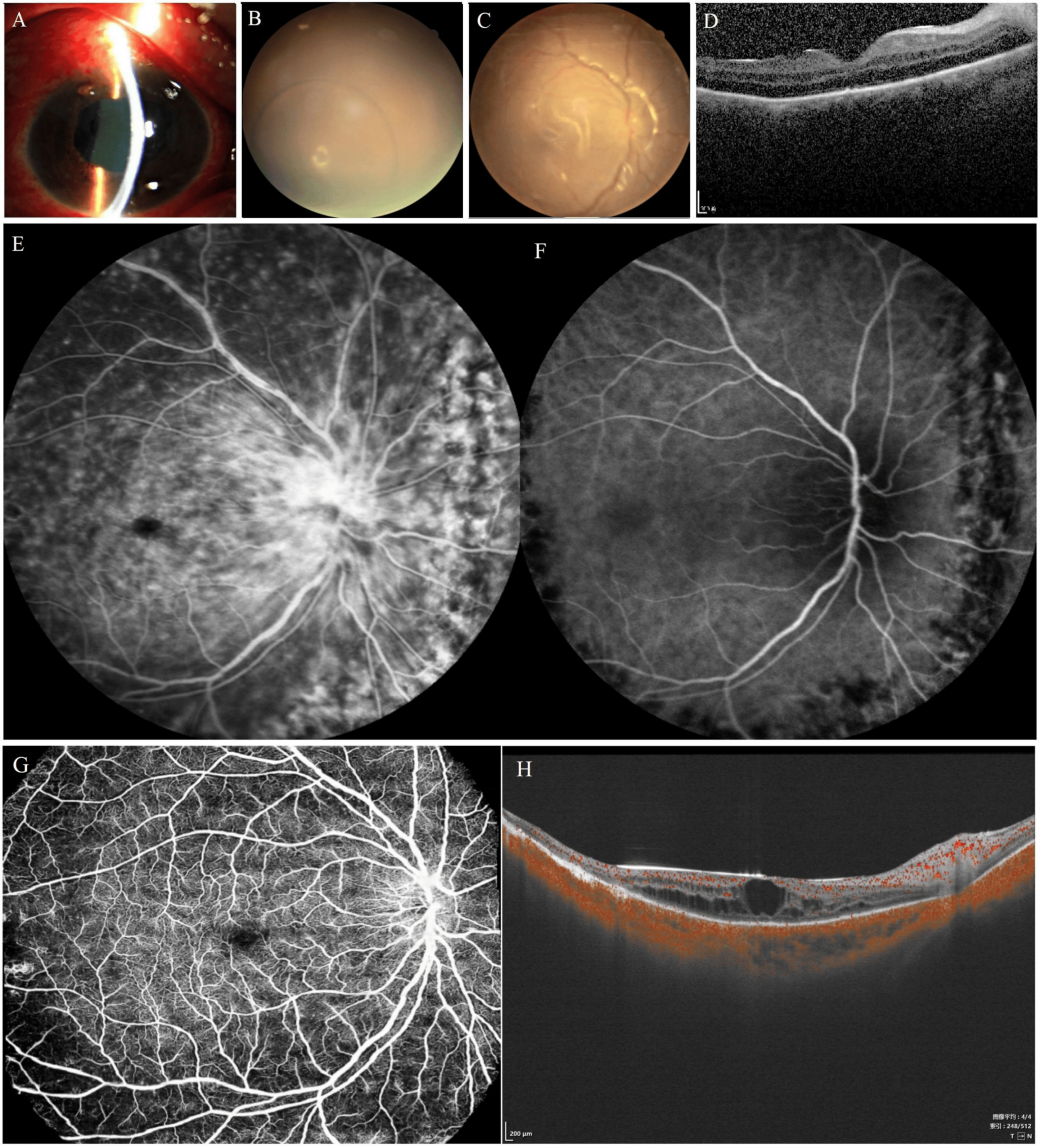
Postoperative status of the right eye after vitreoretinal surgery. (A) Significant exudates remained in the anterior chamber. (B) Voriconazole precipitate (insoluble in silicone oil) within the vitreous cavity due to silicone oil tamponade. (C) Dilated retinal veins seen in the fundus image.  (D) OCT image showed an intact macular structure with small pinpoint hyperreflective foci on the retinal pigment epithelium (RPE). (E-F) Composite of synchronous FFA and ICGA at the three-month postoperative follow-up. The FFA late phase (E) demonstrates extensive scattered leakage of RPE. The concurrent ICGA (F) shows an absence of choroidal vascular abnormalities. The well-demarcated hypofluorescent areas (black) correspond to previous retinal laser photocoagulation spots. (G-H) Two-year follow-up (one and a half years after discontinuation of oral antifungals) using swept-source OCT angiography (SS-OCTA) showed mild retinal venous dilation and macular edema.

One day postoperatively, the VA in the right eye was hand motion (HM). It improved to counting fingers (CF) at five days post-op. Significant exudates remained in the anterior chamber (Figure 4A). Due to the silicone oil tamponade, voriconazole precipitates (which are insoluble in silicone oil) were visible within the vitreous cavity (Figure 4B). Retinal venous dilation was observed (Figure 4C). OCT revealed an intact macular structure with small, pinpoint hyperreflective foci on the retinal pigment epithelium (RPE) (Figure 4D). Simultaneous fundus fluorescein angiography (FFA) and indocyanine green angiography (ICGA) performed three months postoperatively showed numerous, variably sized multifocal RPE leakage sites in the right eye (Figure 4E). At the two-year follow-up, eighteen months after cessation of oral antifungal therapy, swept-source OCT angiography (SS-OCTA) indicated mild retinal venous dilation, macular edema, and the development of an epiretinal membrane in the right eye (Figure 4F). No toxic effects of the drug were found during the whole course of treatment and during the two-year follow-up.

**Figure 5:**
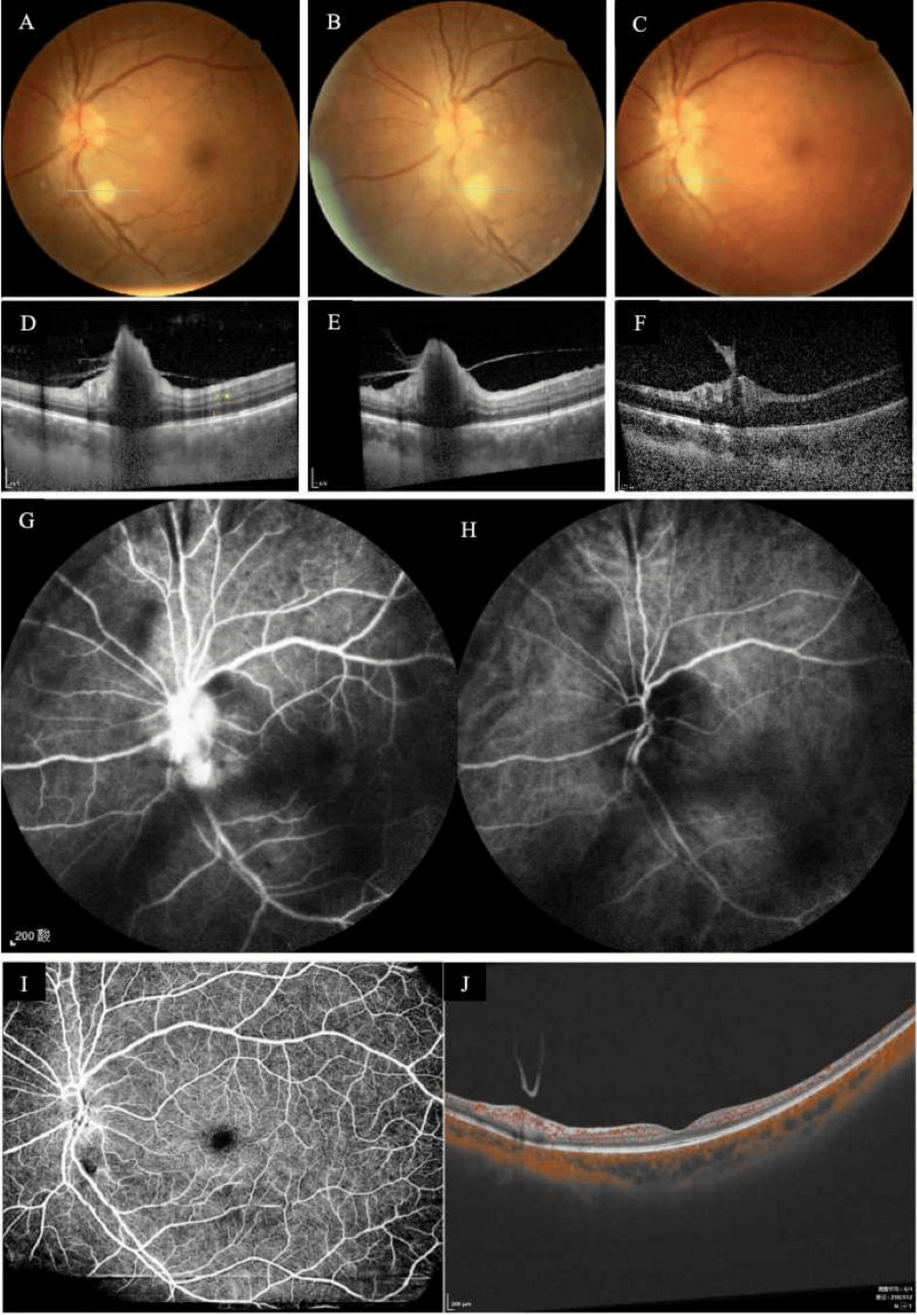
Follow-up after intravitreal antifungal injections in the left eye. Serial OCT B-scans obtained 10, 20, and 40 days after the initial intravitreal injection (A, B, C) demonstrate progressive detachment of the superficial retinal fungal infiltrate into the vitreous cavity. (D-E) Concurrent fluorescein angiography (FFA) and indocyanine green angiography (ICGA) at the three-month post-treatment follow-up. The FFA reveals vitreous opacities and fluorescein leakage from the optic disc and the area inferior to it. The corresponding ICGA shows no evidence of choroidal vascular abnormalities. (F-G) At the two-year follow-up, no significant retinal vascular or structural abnormalities were observed. The vitreous cavity was clear, indicating complete resolution of inflammation. A membranous structure floating anterior to the optic disc exhibited reflectivity similar to the spiky membrane noted over the retinal surface at baseline but displayed a smoother contour, resembling a detached posterior vitreous cortex.

The vision acuity in the left eye gradually recovered from 20/50 to 20/35. Sequential follow-up fundus color photographs (Figures 5A-5C) and OCT B-scan images of the corresponding location of the light blue positioning line (Figures 5D-5F) obtained 10, 20, and 40 days after the initial intravitreal injection showed that the superficial retinal fungal infiltration of the left eye gradually detached into the vitreous cavity. However, the patient consistently reported increased floaters. FFA at three months post-treatment showed persistent fluorescent leakage in optic disc vessels and lesions inferior to the optic disc (Figure 5G). At the two-year review, the retinal vasculature and structure of the left eye were unremarkable, with a clear vitreous cavity, indicating complete resolution of inflammation. The fibrous and spicule-like membranes on the retinal surface observed at baseline showed similar reflectivity but a smoother morphology on OCT, resembling the detached posterior hyaloid cortex (Figure 5H). 

## Discussion

This report presents a rare case of bilateral endogenous Aspergillus fumigatus endophthalmitis in a previously healthy 50-year-old woman following eyebrow tattooing. The clinical course, therapeutic response, and imaging evolution provide valuable insights into the pathogenesis, diagnostic challenges, and management strategies for endogenous fungal endophthalmitis.

Firstly, the most striking feature of this case is the absence of traditional immunosuppression or underlying comorbidities, which contrasts with the well-established predisposition of endogenous fungal endophthalmitis in immunocompromised hosts [4,5]. The infection was highly suspected to originate from the recent facial tattooing procedure [6-8]. The plant-based dyes or instruments used could have been contaminated with environmental fungi (Aspergillus species are ubiquitous in soil and organic matter) [5], and the microtrauma created by the tattooing needle potentially provided a direct portal of entry for this opportunistic pathogen (confirmed by metagenomic sequencing as A. fumigatus) into the bloodstream, leading to subsequent hematogenous dissemination to the eyes. This mechanism shares similarities with the case reported by Huang and Yuan, which emphasized that in trauma patients with concurrent systemic infection (e.g., Staphylococcus aureus bacteremia), fundus manifestations might be misdiagnosed as non-infectious retinopathy when they could indeed represent hematogenous endophthalmitis [4]. Consequently, invasive cosmetic procedures, including eyebrow tattooing and body art, should be recognized as a non-negligible potential risk factor for endogenous endophthalmitis, and clinicians should maintain a high index of suspicion [9].

Secondly, this case starkly illustrates the severe consequences of initial misdiagnosis and inappropriate corticosteroid administration. The patient was initially misdiagnosed with "iritis" due to anterior chamber inflammation and treated with systemic and topical corticosteroids. While steroids suppress inflammation, their immunosuppressive effects profoundly compromise local host defenses, potentially leading to rampant exacerbation of fungal infection. This aligns perfectly with the principle emphasized by Rajendran et al. in their case of Fusarium endophthalmitis: avoiding corticosteroid use before excluding infection [10]. This course serves as a critical warning that infectious etiologies must be high on the differential diagnosis list for any "atypical uveitis," especially in patients with a recent history of trauma or medical procedures [11]. Early and aggressive etiological investigation, such as aqueous or vitreous sampling for molecular testing or culture, is paramount to avoid misdiagnosis [10,12]. The study by Durand et al. also demonstrates that for exogenous fungal endophthalmitis (e.g., post-operative), prompt and accurate pathogen identification and subsequent targeted therapy (e.g., with voriconazole) are crucial for salvaging vision [13].

Regarding management, this case employed a combination strategy including PPV, silicone oil tamponade, and intraocular and systemic antifungal therapy (with voriconazole as the cornerstone). Voriconazole has become a first-line agent for fungal endophthalmitis [10,12,13] due to its excellent intraocular penetration (vitreous concentrations can reach 38% of plasma levels after oral administration) and its efficacy against a broad spectrum of fungi, including Aspergillus and Fusarium species [10,12]. In this case, the significant visual recovery in the left eye and the eventual control of infection in the right eye (despite a poor visual prognosis due to the severity of the initial presentation) following multiple bilateral intravitreal voriconazole injections corroborate its effectiveness in treating invasive fungal intraocular infections [10,12]. Notably, Weishaar et al., in their study on endogenous Aspergillus endophthalmitis, stated that visual prognosis often remains poor if the macula is involved, even after aggressive vitrectomy and intravitreal amphotericin B [12], which is consistent with the ultimate outcome in the patient's right eye.

Furthermore, this case provided a rare dynamic perspective on the resolution of fungal endophthalmitis through serial imaging. Sequential OCT follow-up of the fellow eye (left eye) clearly documented the entire process whereby the fungal infiltrate in the inner retina gradually detached and dissipated into the vitreous cavity. This vivid imaging evolution not only demonstrates the effectiveness of antifungal treatment but also provides objective evidence for assessing therapeutic response and prognosis. Reginatto et al., in their review on ocular fungal infections, also emphasized the critical role of multimodal imaging, including OCT and FFA, in evaluating the extent of infection, monitoring treatment response, and identifying complications [5].

However, it is crucial to acknowledge that despite aggressive surgical and medical intervention, the delay in initial diagnosis and the inappropriate use of corticosteroids ultimately resulted in permanent visual impairment in the right eye (cystoid macular edema, epiretinal membrane formation). This underscores the profound and long-lasting damage severe endophthalmitis can inflict on retinal architecture. As widely noted in the literature, successful outcomes heavily depend on early diagnosis and the timely initiation of targeted antifungal therapy [5,10,12].

## Conclusions

This case demonstrates that invasive cosmetic procedures, such as eyebrow tattooing, can serve as a portal of entry for opportunistic fungi like Aspergillus fumigatus, potentially triggering severe bilateral endogenous endophthalmitis even in immunocompetent individuals. Initial misdiagnosis as non-infectious iritis and subsequent corticosteroid therapy significantly exacerbated the infection. This underscores the critical importance of prioritizing infectious etiologies, particularly fungal, in the differential diagnosis of any atypical uveitis, especially in patients with a recent history of trauma or procedures. Early vitreous sampling and the application of molecular diagnostic techniques like metagenomics are important for achieving a precise diagnosis and enabling the timely initiation of targeted antifungal therapy. The combination of intravitreal and systemic voriconazole constitutes an effective core strategy for managing such infections. Serial OCT imaging provides an invaluable tool for the non-invasive monitoring of lesion dynamics and treatment response. Despite aggressive intervention, diagnostic delays can still lead to irreversible visual impairment, highlighting the necessity for heightened clinical vigilance, detailed history-taking, and public education regarding the infectious risks associated with cosmetic procedures.
